# In vivo tumour imaging employing regional delivery of novel gallium radiolabelled polymer composites

**DOI:** 10.1186/s40824-021-00210-0

**Published:** 2021-03-31

**Authors:** Ross W. Stephens, Gregory D. Tredwell, Jessica L. Bell, Karen J. Knox, Lee A. Philip, Tim J. Senden, Michael J. Tapner, Stephanie A. Bickley, Marcel R. Tanudji, Stephen K. Jones

**Affiliations:** 1 The Biomedical Radiochemistry Laboratory, Department of Applied Mathematics, Research School of Physics, Australian National University, Canberra, ACT Australia; 2 Sirtex Medical Ltd, Sydney, Australia

**Keywords:** Vascular particle delivery, Tumour plexus, Tumour imaging, Radiolabelled polymer microspheres, Rabbit tumour model

## Abstract

**Background:**

Understanding the regional vascular delivery of particles to tumour sites is a prerequisite for developing new diagnostic and therapeutic composites for treatment of oncology patients. We describe a novel imageable ^67^Ga-radiolabelled polymer composite that is biocompatible in an animal tumour model and can be used for preclinical imaging investigations of the transit of different sized particles through arterial networks of normal and tumour-bearing organs.

**Results:**

Radiolabelling of polymer microspheres with ^67^Ga was achieved using a simple mix and wash method, with tannic acid as an immobilising agent. Final in vitro binding yields after autoclaving averaged 94.7%. In vivo stability of the composite was demonstrated in New Zealand white rabbits by intravenous administration, and intrahepatic artery instillations were made in normal and VX2 tumour implanted rabbit livers. Stability of radiolabel was sufficient for rabbit lung and liver imaging over at least 3 hours and 1 hour respectively, with lung retention of radiolabel over 91%, and retention in both normal and VX2 implanted livers of over 95%. SPECT-CT imaging of anaesthetised animals and planar imaging of excised livers showed visible accumulation of radiolabel in tumours. Importantly, microsphere administration and complete liver dispersal was more easily achieved with 8 μm diameter MS than with 30 μm MS, and the smaller microspheres provided more distinct and localised tumour imaging.

**Conclusion:**

This method of producing ^67^Ga-radiolabelled polymer microspheres is suitable for SPECT-CT imaging of the regional vascular delivery of microspheres to tumour sites in animal models. Sharper distinction of model tumours from normal liver was obtained with smaller MS, and tumour resolution may be further improved by the use of ^68^Ga instead of ^67^Ga, to enable PET imaging.

## Background

Understanding the regional vascular delivery of particles to tumour sites is a prerequisite for developing new diagnostic and therapeutic composites for treatment of oncology patients. Clearly this is important in order to predict organ distribution prior to any treatment with particles carrying cytotoxic radioisotopes or toxins. This applies especially to radioisotope treatments with longer half-lives emitting relatively high energy beta emission, such as in selective internal radiation therapy (SIRT) of metastatic colorectal tumours present in the liver [1–3].

We have previously demonstrated liver tumour imaging in animal models using cationised carbon nanoparticles, comprised of a core of ^99m^Tc encapsulated in graphitic carbon, as a stable electrostatic radiolabel for polymer microspheres (MS) [4]. However, the Gallium isotopes already in clinical use have significant advantages, offering both single-photon emission computed tomography (SPECT) imaging with ^67^Ga and higher resolution positron emission tomography (PET) imaging with ^68^Ga. Gallium-67 has a physical half-life of 78.3 h and decays by electron capture, emitting gamma radiation suitable for SPECT imaging. The soluble citrate salt of ^67^Ga is currently used clinically as an intravenous injection for imaging the extent of Hodgkin’s disease, lymphomas and bronchogenic carcinoma [5]. Gallium-68 is a positron emitter with a 1.13 h half-life, but despite this short half-life, the radioisotope can be very useful for PET imaging when produced at the point of use from a ^68^Ge/^68^Ga generator [6–8].

In this report we describe a novel composite comprising ^67^Ga immobilised on a polymer surface with tannic acid (TA), that has favourable stability in vitro and in vivo for preclinical animal imaging studies. Using SPECT-CT of intact animals, and planar imaging of excised livers, we demonstrate accumulation of MS at tumour sites in livers following arterial administration. We show that tumour accumulation is related to MS size, with smaller 8 μm MS producing superior tumour imaging to 30 μm MS.

## Results

### Synthesis of ^67^GaTA-MS and in vitro stability to autoclaving

**Fig. 1:**
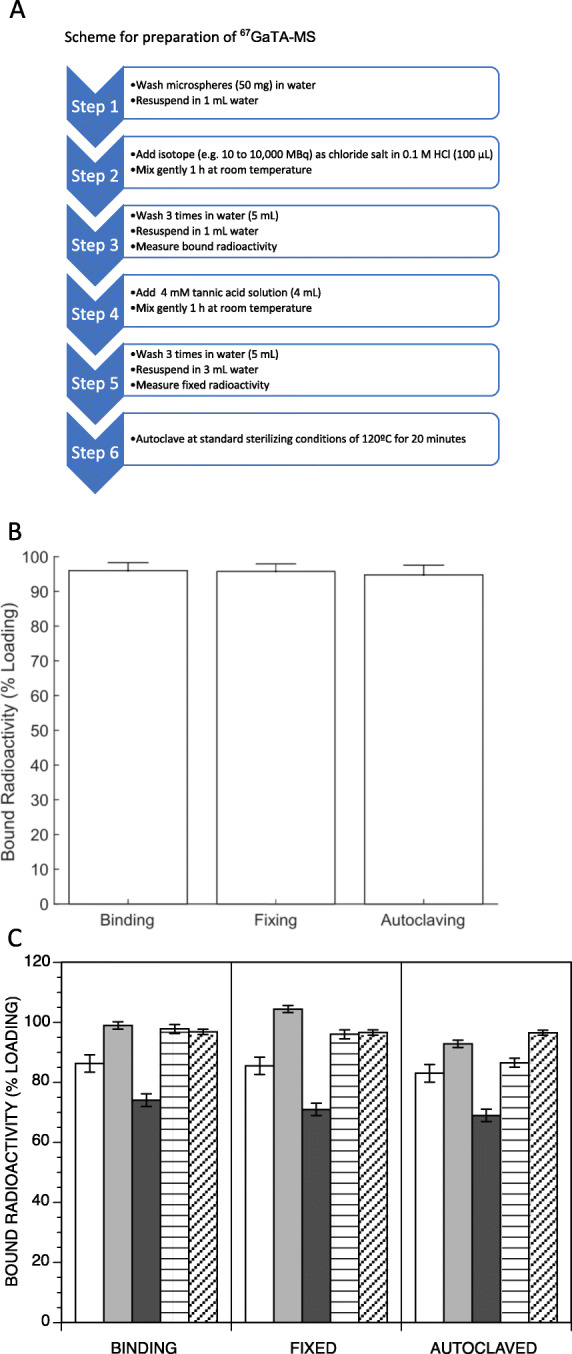
Radiolabelling method for polymer MS. **a** Step by step scheme for the preparation of ^67^GaTA-MS, starting from an example amount of 50 mg of sulfonated polystyrene MS. **b** Optimised radiolabelling binding efficiency of ^67^Ga on both 8 μm and 30 μm diameter MS (*n* = 12, *n* = 5, respectively. Data combined). The bound fraction of each isotope loading is shown after the initial binding step (Binding), after the step with TA treatment (Fixed) and after autoclaving (Autoclaved); **c** A comparison of unoptimized microsphere radiolabelling binding efficiency for five different radionuclides. Polystyrene sulfonate MS (50 mg, 30 μm median diameter) was radiolabelled with (columns from left to right) ^67^Ga (100–200 MBq; *n* = 14), In-111 (34 MBq; *n* = 2), Tl-201 (93 MBq; n = 2), Lu-177 (50–150 MBq; *n* = 7) and Y-90 (42 MBq; n = 2). The bound fraction of each radionuclide loading is shown after the initial binding step (Binding), after the step with TA treatment (Fixed) and after autoclaving (Autoclaved)

Polystyrene sulfonate MS of the same type as used in clinical SIRT [2] were labelled with the radioisotope ^67^Ga were prepared in a simple, convenient, mix and wash method utilising TA as a stable complexing agent (Fig. 1A). With the strong anion exchange properties of the polystyrene sulfonate, it was straight forward to bind either the chloride or citrate salts of ^67^Ga in high yields, by mixing with the polymer microsphere in the presence of HCl (0.2 M final concentration). The MS were mixed with the radioisotope at room temperature and then washed 3 times with water using centrifugation to collect the MS. The radioisotope was then fixed by mixing with TA (3 mM) at room temperature for 1 h, followed by a further washing step. The MS could then be autoclaved under standard conditions. The bound radioactivity on the MS following each of the binding, fixing and autoclaving steps was high (Fig. 1B), with final yields after autoclaving averaging 94.7% for both 8 and 30 μm diameter MS. Note that this process can also be applied to other metal radioisotopes such as ^111^Indium, ^201^Thallium, ^177^Lutetium, and ^90^Yttrium, with high binding yields (Fig. 1C, unoptimised yields).

### Leach tests of ^67^GaTA-MS stability by entrapment in normal rabbit lungs

**Fig. 2:**
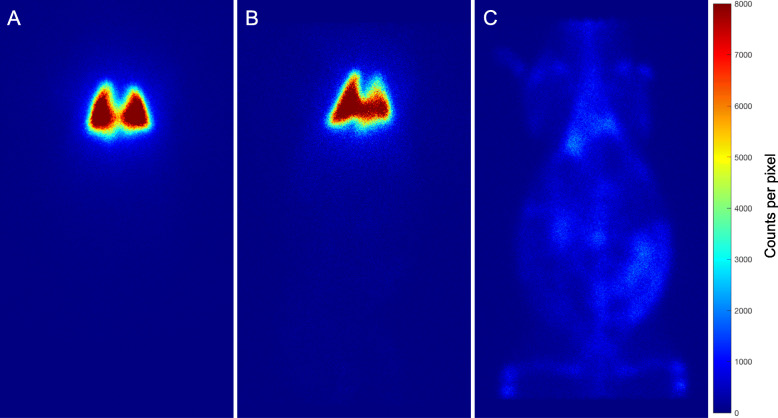
Leach tests of ^67^Ga labelled MS in normal rabbit lungs. Polystyrene sulfonate MS (15 mg, 30 μm and 8 μm median diameter) were radiolabelled with ^67^Ga (avg. 87 MBq) using TA immobilisation. The suspensions in 5% dextrose (5 mL) were injected intravenously (ear vein) into anaesthetised rabbits and planar images of the rabbits were made 3 h post-injection using a GE Hawkeye Infinia SPECT-CT camera with a medium energy collimator and scatter correction. Control rabbits were injected intravenously with soluble ^67^Ga (avg. 87 MBq) for comparison of the biodistribution. Note pronounced lung retention of radiolabel using both ^67^GaTA-MS30 (**a**) and ^67^GaTA-MS8 (**b**), while soluble ^67^Ga citrate was systemically distributed with no lung definition (**c**)

The in vivo stability of the ^67^GaTA-MS preparations was tested by intravenous injection into the lungs of rabbits. Using less than 5 mg MS per kg of body weight, there was no apparent change in breathing over the 3 h post-injection test period. Suspensions of ^67^GaTA-MS30 and ^67^GaTA-MS8 were retained well in the vascular network of the lungs over 3 h, as shown by gamma camera imaging of the anaesthetised animals (Fig. 2A and B, respectively), compared with intravenous injection of soluble ^67^Ga (Fig. 2C). After 3 h the animals were euthanised, and the lungs, livers and body were imaged separately for more quantitative radioactivity biodistribution data (Table 1). For ^67^GaTA-MS30, the average proportion of radioactivity retained in the lungs was 97.3 ± 0.4% of the total body activity, and only 0.61 ± 0.1% was present in the excised liver. For ^67^GaTA-MS8, the average proportion of radioactivity retained in the lungs was slightly lower at 91.1 ± 0.4% of the total body activity, and with 1.5 ± 0.3% present in the liver. For comparison, rabbits injected with soluble ^67^Ga had low lung and liver radioactivity with only 5.3 and 9.9%, respectively, of the total body radioactivity (Fig. 2C and Table 1).

**Table 1:** Leach Tests for ^67^Ga Labelled MS in Rabbit Lungs

Isotope Form	No. of Tests	Lungs [%Total]	Liver [%Total]	Body [%Total]
MS30	5	97.3 ± 0.4	0.61 ± 0.1	2.5 ± 0.3
MS8	3	91.1 ± 0.4	1.5 ± 0.3	7.3 ± 0.7
Soluble	2	5.3 ± 0.4	9.9 ± 0.7	83.4 ± 0.3

Polystyrene sulfonate MS (15 mg, 30 μm and 8 μm median diameter) were radiolabelled with ^67^Ga (avg. 87 MBq) using TA immobilisation. Suspensions of the MS in 5% dextrose (5 mL) were injected intravenously (ear vein) into anaesthetised rabbits. Intravenous injection of soluble ^67^Ga (avg. 87 MBq) was also performed to provide a comparison of the biodistribution. The rabbits were euthanized after 3 h for dissection and gamma camera imaging of lungs, liver, and body. The activities of all images were corrected for background and the activity of each organ expressed as a percentage of the total activity

### Biodistribution of ^67^GaTA-MS in normal rabbit livers

**Fig. 3:**
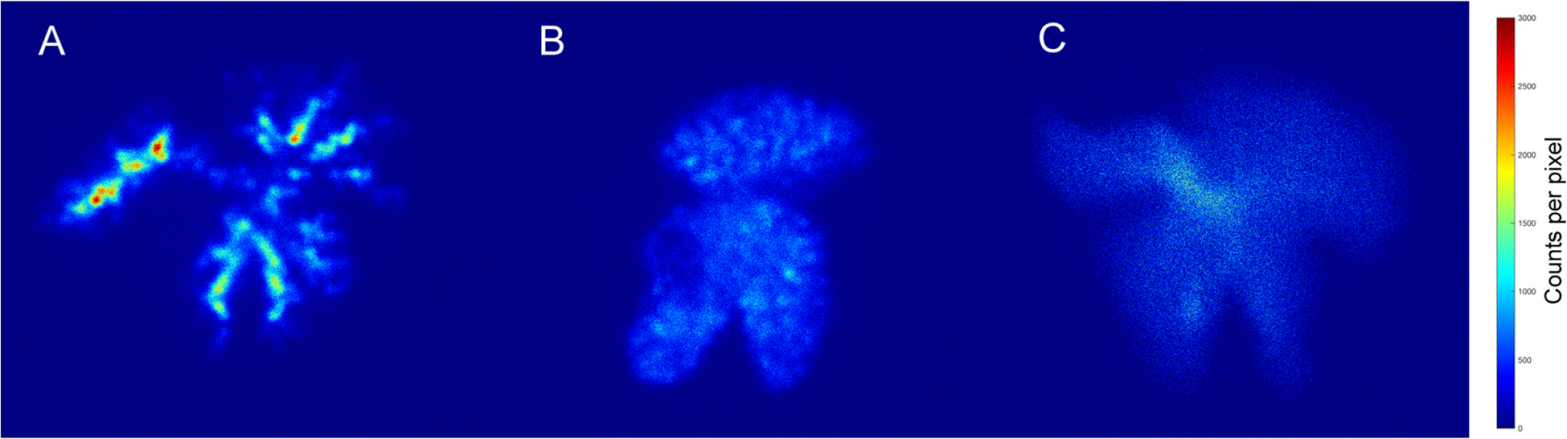
Retention of ^67^Ga labelled MS in normal rabbit livers. Polystyrene sulfonate MS (40 mg, 30 μm and 8 μm median diameter) were radiolabelled with ^67^Ga (avg. 110 MBq) using TA immobilisation. The suspensions in 5% dextrose (5 mL) were instilled into the hepatic artery of anaesthetised rabbits and planar images of excised livers were made 1 h post-injection using a GE Hawkeye Infinia SPECT/CT camera with a medium energy collimator and scatter correction. Soluble ^67^Ga citrate (avg. 110 MBq) was instilled into hepatic arteries of control rabbits for comparison with the retention of microspheres. Note the prominent vessel features and less extensive volume filling of the liver obtained with ^67^GaTA-MS30 (**a**), compared to the more extensively dispersed fine network obtained with ^67^GaTA-MS8 (**b**). Under the same conditions, soluble ^67^Ga citrate was mostly purged from the liver by the normal blood perfusion (**c**)

Suspensions of both the ^67^GaTA-MS30 and ^67^GaTA-MS8 were administered to rabbit livers via the hepatic artery. Some administration failures were encountered with the larger ^67^GaTA-MS30, when the MS remained predominantly at a central part of the liver and did not disperse widely in the organ. There were no administration failures when using the smaller ^67^GaTA-MS8. Planar gamma imaging of anaesthetised animals showed virtually complete retention of the radiolabel in the liver, and after 1 h following administration the animals were euthanised and the lungs and liver were dissected to obtain more quantitative radioactivity biodistribution (Table 2). Normal rabbit liver radioactivity when instilled with the ^67^GaTA-MS30 composites was an average 98.9 ± 0.3% of the total body radioactivity, and lung activity was only 0.07 ± 0.03%. With ^67^GaTA-MS8, the mean liver activity was slightly lower at 95.2 ± 2.4%, and the lung activity 0.57 ± 0.35% of the total body radioactivity. In comparison, soluble ^67^Ga displayed no appreciable affinity for either the liver or lungs, with mean radioactivity of 15.4 and 4.7%, respectively, of the total body radioactivity. Images of the excised livers revealed a pronounced difference in the distribution for the 2 different diameters of MS, ^67^GaTA-MS30 and ^67^GaTA-MS8 (Fig. 3A and B, respectively). Livers instilled with ^67^GaTA-MS30 did not have radioactivity extending throughout the organ, but instead it appeared to have been arrested at limiting diameters of the arterial network, displaying a course tree-like structure. The image shown in Fig. 3A was the best liver dispersal obtained for ^67^GaTA-MS30. In contrast, the smaller diameter ^67^GaTA-MS8 routinely dispersed well within the liver and displayed a much finer liver distribution (Fig. 3B), being able to disperse to the finer arterial vessels throughout the whole tissue, while still not being able to pass through the microcapillaries to the venous collection. The soluble ^67^Ga instilled intra-arterially in the liver displayed no appreciable retention in the liver, and was distributed systemically throughout the rabbit (Fig. 3C and Table 2).

**Table 2:** Retention of ^67^Ga Labelled MS in Rabbit Livers with and without Implants of VX2 tumours

Organ Test	MS Diameter [μm]	No. of Tests	Lungs [%Total]	Liver [%Total]	Body [%Total]
Normal Liver	30	3	0.07 ± 0.03	98.9 ± 0.3	0.97 ± 0.23
	8	2	0.57 ± 0.35	95.2 ± 2.4	4.1 ± 2.0
	Soluble	2	4.7 ± 0	15.4 ± 3.0	78.1 ± 3.2
VX2 Liver	30	2	0.02 ± 0.03	99.2 ± 0.2	0.78 ± 0.18
	8	4	0.62 ± 0.07	95.7 ± 0.5	3.5 ± 0.5
	Soluble	2	1.83 ± 0.13	4.5 ± 1.0	93.1 ± 0.95

Polystyrene sulfonate MS (15 mg, 30 μm and 8 μm median diameter) were radiolabelled with ^67^Ga (avg. 110 MBq) using TA immobilisation. Suspensions of the MS in 5% dextrose (5 mL) were instilled into the hepatic artery of anaesthetised rabbits. Instillation of soluble ^67^Ga (avg. 110 MBq) was also performed to provide a comparison of the biodistribution. The rabbits were euthanized after 1 h for dissection and gamma camera imaging of lungs, liver, and body. The activities of all images were corrected for background and the activity of each organ expressed as a percentage of the total activity. Note pronounced liver retention of radiolabel using both ^67^GaTA-MS30 and ^67^GaTA-MS8 compared to liver retention of soluble ^67^Ga. Retention of microsphere radiolabel in livers with VX2 tumour implants was indistinguishable from retention by normal livers

### Biodistribution of ^67^GaTA-MS in rabbit livers with implanted VX2 tumours

**Fig. 4:**
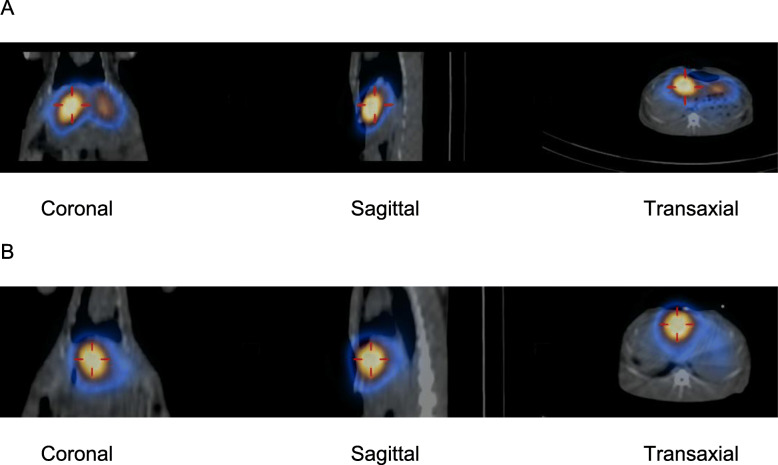
SPECT-CT of rabbits with VX2 liver tumours using ^67^Ga labelled MS. Polystyrene sulfonate MS (40 mg, 30 μm and 8 μm median diameter) were radiolabelled with ^67^Ga (avg. 110 MBq) using TA immobilisation. The suspensions in 5% dextrose (5 mL) were instilled into the hepatic artery of anaesthetised rabbits bearing VX2 tumour implants in their livers. SPECT-CT imaging of the rabbits was performed 1 h post-injection using a GE Hawkeye Infinia SPECT/CT camera. Hybrid SPECT-CT summary views are shown for ^67^GaTA-MS30 (**a**) and ^67^GaTA-MS8 (**b**). In (**a**) note the ^67^GaTA-MS30 apparent at the tumour site in the liver’s right lobe (left side of the coronal image and left side of the transaxial image), but significant activity also apparent in the rest of the liver. In (**b**), the ^67^GaTA-MS8 shows more selective accumulation of radioactivity at the tumour site in the implanted lobe, with only low activity in the rest of the liver

Implantation of rabbit livers with small pieces of VX2 tumours was performed using sterile keyhole surgery on a single site on one lobe, and after 18–21 days of growth, tumours appeared macroscopically on dissection as a single oblate ellipsoid of up to 2 cm diameter. Intraarterial instillations of ^67^GaTA-MS30 and ^67^GaTA-MS8 were then made in these rabbit livers hosting grown VX2 tumour implants. SPECT-CT imaging of the intact animals showed good retention of both sizes of MS in the liver (Fig. 4). Within the liver, ^67^GaTA-MS30 showed isotope activity mainly in the right lobe hosting the tumour, as seen in the left side of the coronal image and in the left side of the transaxial image in Fig. 4A. However, there was significant activity in the rest of the liver, as seen in the right side of the coronal image and in the right side of the transaxial image in Fig. 4A. Importantly, using the smaller ^67^GaTA-MS8, accumulation of isotope appeared more localised to the single tumour site in the implanted lobe, as seen in the corresponding images of Fig. 4B.

**Fig. 5:**
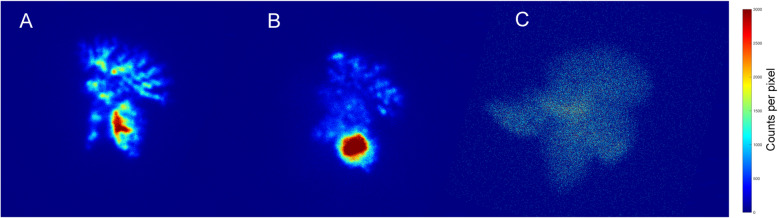
Planar imaging of excised livers with VX2 tumours. The same rabbits shown in Fig. 4A and B above were dissected to enable planar imaging of their livers. As in Fig. 3 above for normal livers, note the prominent vessel features and less extensive volume filling of the liver obtained with ^67^GaTA-MS30 (**a**), compared to the more extensively dispersed fine network obtained with ^67^GaTA-MS8 (**b**). Importantly, while the tumour site in (**a**) was identifiable, having increased activity compared to the rest of the liver, the use of ^67^GaTA-MS8 in (**b**) revealed a more sharply distinct tumour site of high activity, with only weak activity in the rest of the liver. For comparison, the excised liver from a rabbit instilled with soluble ^67^Ga citrate showed extensive loss of isotope from the organ, and although a tumour was present in this liver, there was no appreciable tumour retention of isotope (**c**)

This result was confirmed by excising the livers from the same rabbits and making planar images, when it was apparent that while both ^67^GaTA-MS30 and ^67^GaTA-MS8 displayed a noticeable accumulation of the label at the tumour site (Fig. 5A and B respectively), the smaller diameter ^67^GaTA-MS8 had a more pronounced tumour accumulation, appearing in planar imaging as a sharply defined circular feature of high activity (Fig. 5B). In lobes that did not contain the VX2 tumour, ^67^GaTA-MS30 displayed a coarse tree-like structure for the distribution of the radiolabel, and some normal liver tissue areas had significant radioactivity (Fig. 5A). By contrast, livers instilled with the smaller ^67^GaTA-MS8 had lower intensities of radioactivity in normal lobes Fig. 5B), presumably due to volumetric dilution by more extensive dispersal throughout the tissue via finer blood vessels. Importantly, this was not accompanied by escape of the ^67^GaTA-MS8 from VX2 implanted livers into the systemic circulation; 1 h following intraarterial instillation 95.2 ± 2.4% of the total body radioactivity was still present in the liver (Table 2). By contrast, instillation of soluble ^67^Ga did not produce any discernible liver or tumour retention, with only 4.5 ± 1.0% of the total body radioactivity remaining in the organ (Fig. 5C and Table 2).

## Discussion

This article describes a novel method for radiolabelling polymer MS with Gallium isotopes, employing a simple, convenient mix and wash method requiring no special equipment. A product with high radiopurity that is stable to autoclave sterilisation can be obtained with minimal handling. The product has utility in imaging regional vascular delivery of polymer MS to tumour sites, as demonstrated here in a rabbit liver tumour model. Preliminary indications of biocompatibility were seen in this animal model setting. Importantly, the results show that smaller MS have superior administration, dispersal and tumour imaging properties.

Gallium isotopes can be considered the preferred choice for making imageable polymer particles that can be used to predict biodistribution of the corresponding therapeutic particles in organs hosting tumours. Gallium-67 citrate with its relatively long half-life of 78.3 h is already a current clinical product for SPECT imaging of patients with Hodgkin’s disease [5], lymphomas and bronchogenic carcinoma. In our method, by simply adjusting the pH with 1 M HCl, the ^67^Ga^3+^ ions could be dissociated from the citrate complex and bound readily to polystyrene sulfonate MS, which has the properties of a strong cation exchange resin. A stable radioisotope complex is then simply formed by addition of TA, and the method does not require the use of any special equipment. Clearly, it would be straight-forward to adapt this method to ^68^Ga eluted from a commercially available germanium-68/Gallium-68 generator. The radioisotope eluted with hydrochloric acid could be directly bound to the MS in a single step enabling PET imaging with the final radiolabelled MS. Furthermore, we have applied this method to other metal radioisotopes such as ^111^Indium, ^201^Thallium, ^177^Lutetium, and ^90^Yttrium, with high binding yields.

Tannins, a class of astringent, polyphenolic biomolecules, are major constituents of our beverages and food stuffs [9], so they are known to be biocompatible. The metal ion chelation properties of TA are well known, and the phenolic-metal ion complexes are often coloured [10]. It is believed that the highly stable complexes of Fe (III) with TA are not bioavailable [9], and in fact consumption of large quantities of tannin-rich foods is sometimes associated with iron-deficiency anemia [11].

Free Gallium in the blood acts as an iron analogue, binding to the plasma protein transferrin. A strong interaction between Gallium and TA is therefore likely and important for minimising unwanted organ uptake by interfering with interactions of Gallium with plasma proteins. As was observed for a patient treated with deferoxamine prior to ^67^Ga-citrate scintigraphy demonstrating an altered biodistribution of Gallium [12].

Previously described methods for immobilising radioisotopes such as ^67^Ga have involved the preparation of covalently linked macrocyclic ligands such as DOTA and NOTA. While DOTA is the mostly used chelator, the smaller, tri-chelator, NOTA was found to bind Gallium better [13, 14]. In this method the TA is not covalently bound to the MS, but instead forms a stable complex involving both the Gallium ion and the polystyrene sulfonate surface.

This complex is stable to autoclave conditions and the radiolabelled MS were stable for 3 h in vivo following intravenous delivery to the lungs of New Zealand white rabbits. Furthermore, hepatic artery administration of the ^67^GaTA-MS showed very efficient retention of the radiolabel at limiting diameters of the liver arterial network. As was observed for the Tc99m labelled MS that we described earlier [4], the smaller diameter ^67^GaTA-MS8 displayed a much finer liver distribution, dispersing more extensively into the vascular network than the larger ^67^GaTA-MS30, which tended to define a jagged, tree-like pattern of the larger vessels.

For rabbits with implanted VX2 tumours, there was no significant evidence of hepatopulmonary or hepatogastric shunting, and retention of radiolabel within livers hosting a tumour was maintained at a very high level. Images of livers implanted with VX2 tumours demonstrated that the tumours had remodelled the local arterial supply, producing a bias of distribution and accumulation of radiolabelled MS in tumours, as seen before in computed tomography studies [15]; this was evident for both sizes of MS tested. However, the smaller MS produced a more defined image of tumours, presumably due to its ability to penetrate further into the plexus of abnormally tortuous angiogenic vessels in the tumour microenvironment [16], while still being large enough not to transit to the venous collection and enter the systemic circulation. Our study of 8 μm MS extends further downward the range of MS size reported previously [17], when more extensive imaging of VX2 vascular networks was obtained with 30 μm than with 100 μm MS. It is evident that the tumour plexus, consistent with the observed increase in flow resistance [18], tends to behave as a labyrinthine trap that limits transit of non-deformable polymer particles, even those comparable in size to deformable blood cells. Blood cells, especially erythrocytes, are well known [19] to traverse capillaries by deforming to a smaller cross-section, whereas non-deformable particles of similar size to blood cells will not traverse capillaries.

## Conclusion

We have now demonstrated tumour imaging results for different sizes of 2 types of polymer MS preparations, radiolabelled by the use of 2 completely different labelling chemistries and 2 different radioisotopes, i.e. ^99m^Tc-nanoparticle radiolabelled MS [4] and ^67^GaTA-MS. The consistent message is that smaller MS, even down to 8 μm diameter, produce better tumour definition in imaging, and without appreciable leakage to the systemic circulation. The increased ability of smaller radiolabelled MS to highlight tumours in imaging is informative for scoping the location, size and number of tumours in an organ, and potentially also useful as an indicator of reduced tumour perfusion achieved by angiogenesis inhibitors. All these results assist in evaluating accessibility of solid tumours to therapeutic MS and the likelihood of achieving a therapeutic impact. Considerable further work is required however at the preclinical level to specifically address the biocompatibility, toxicology and clearance of this novel composite.

## Materials and methods

### Stable labelling of MS with ^67^Ga

Polystyrene sulfonate MS of the same type as used in clinical SIRT [2] and of two different sizes were used in this study; 30 μm (MS30), and 8 μm (MS8) median diameter (Sirtex Medical Ltd., Sydney). The radioisotope ^67^Ga was obtained as an aqueous citrate salt from Global Medical Solutions Australia. USP grade TA was purchased from Sigma-Aldrich (Sydney, Australia). Radiolabelled ^67^Ga MS were prepared as previously described [20] and summarised in Fig. 1A. Briefly, MS (15–40 mg) were first washed 3 times with water (5 mL) using centrifugation at 350 g for 2 min. The MS were then mixed for 1 h with ^67^Ga-citrate and 1 M HCl (1 mL) in a final volume of 5 mL water. After washing 3 times using centrifugation (350 g, 2 min), the MS were then resuspended in 3 mM TA (5 mL) for 1 h. After 3 further washes with water, the MS were resuspended in water (3 mL) and autoclaved at 120 °C for 20 min to give the radiolabelled product.

### Leach tests of ^67^Ga labelled MS (^67^GaTA-MS) in normal rabbit lungs

All rabbit procedures adhered to the National Health and Medical Research Council’s Australian code for the care and use of animals for scientific purposes (Australian Government, 8th Ed., 2013), and the experimental protocols were approved by the Australian National University (ANU) Animal Ethics Committee. Rabbit imaging studies were carried out using intubation to deliver ventilation anaesthesia with isoflurane, so that biodistribution of the radiolabel in live animals could be followed for up to 3 h. Imaging of the anaesthetised rabbits, and their excised organs after 3 h, was performed with a Hawkeye Infinia SPECT-CT camera (GE Healthcare). Images were obtained using a medium energy collimator with energy emission windows of 93 ± 13, 184 ± 10, and 300 ± 10 keV, and Compton scatter correction windows of 75 ± 7, and 140 ± 10 keV. To test the lung retention of the radiolabelled MS, suspensions of ^67^GaTA-MS30 and ^67^GaTA-MS8 (avg. 87 MBq on 15 mg MS in 5 mL 5% dextrose) were injected intravenously into an ear vein, so that the MS were mechanically arrested at limiting diameters in the arterial network of the lungs. Static 5 min acquisitions were made on a 1024 × 1024 matrix, approx. 10 min after injection of the MS and again every hour up to 3 h, before the rabbits were euthanized by lethal injection while still under anaesthesia. The lungs and liver were then excised after tying off blood vessels to prevent leakage of the radioisotope, and the excised organs were imaged separately using a 5 min acquisition on a 1024 × 1024 matrix and utilising the camera’s zoom function (4×). Counts registered in the acquisitions were corrected for the background activity of the corresponding field, and the corrected counts were used for calculation of the percentage activity in the lungs, liver, and body. The DICOM image files were read into MATLAB (MathWorks, MA, USA) and were scaled such that the total counts of individual images were equal.

### Arterial distribution of ^67^GaTA-MS in normal rabbit livers

Intrahepatic artery instillations of ^67^GaTA-MS30, ^67^GaTA-MS8 (40 mg each) and soluble ^67^Ga-citrate were performed by catheterisation of the cystic artery and using pulses of the particle suspension (total 5 mL 5% dextrose, avg. 110 MBq) interspaced with normal hepatic artery blood flow, so as to disperse the radiolabelled material throughout the liver with close to normal arterial blood perfusion conditions [21]. The MS were kept suspended by gentle agitation during instillation. Static 5 min acquisitions were made on a 1024 × 1024 matrix, approx. 10 min after administration of the imaging agent and again after 1 hour, before the rabbits were euthanised by lethal injection while still under anaesthesia. The lungs and liver were excised after tying off blood vessels to prevent leakage of the radioisotope, and the excised organs were imaged separately using a 5 min acquisition on a 1024 × 1024 matrix and utilising the zoom function. Counts registered in the acquisitions were corrected for the background activity of the corresponding field, and the corrected counts were used for calculation of the percentage activity in the organs. The DICOM image files were read into MATLAB (MathWorks, MA, USA) and for each of the images the individual pixel counts were divided by the total image counts and then scaled by a constant factor.

### Imaging of the rabbit VX2 liver tumour model

The transplantable rabbit VX2 tumour [22] was a kind gift of Dr. J Geschwind (Johns Hopkins University, Baltimore, USA) and was maintained as a serial transplant on the hind limbs of New Zealand white rabbits. Liver implants of small pieces of tumour tissue were made at a single site in one lobe by keyhole surgery under ventilation anaesthesia with isoflurane and allowed to grow for 18–21 days before use of the rabbit in imaging experiments. At this stage of growth, the tumour was an oblate ellipsoid of maximum diameter 2 cm, still contained within the liver lobe and not involving the body wall or other organs. Macroscopically, the tumour usually had a white necrotic centre, surrounded by a prominently vascularised peripheral growth zone. Intrahepatic artery instillations of ^67^GaTA-MS30 and ^67^GaTA-MS8 (40 mg each) were made in the livers hosting tumours as above and SPECT-CT imaging with a Hawkeye Infinia camera was performed 1 h post instillation, before the rabbits were euthanised by lethal injection while still under anaesthesia. The lungs and liver were excised after tying off blood vessels to prevent leakage of the radioisotope, and planar images of the excised organs were made separately using a 5 min acquisition on a 1024 × 1024 matrix and utilising the zoom function. Counts registered in the acquisitions were corrected for the background activity of the corresponding field, and the corrected counts were used for calculation of the percentage activity in the organs. The DICOM image files were read into MATLAB (MathWorks, MA, USA) and for each of the images the individual pixel counts were divided by the total image counts and then scaled by a constant factor.

## Data Availability

The datasets generated during and/or analysed during the current study are available from the corresponding author on reasonable request.

## Abbreviations

MS: Polystyrene sulfonate microsphere
PET: Positron emission tomography
SIRT: Selective internal radiation therapy
SPECT: Single-photon emission computed tomography
TA: Tannic acid
^67^GaTA-MS8: ^67^Ga labelled MS of 8 μm diameter
^67^GaTA-MS30: ^67^Ga labelled MS of 30 μm diameter
